# Salt-Tolerant Plants, Halophytes, as Renewable Natural Resources for Cancer Prevention and Treatment: Roles of Phenolics and Flavonoids in Immunomodulation and Suppression of Oxidative Stress towards Cancer Management

**DOI:** 10.3390/ijms24065171

**Published:** 2023-03-08

**Authors:** Hamdoon A. Mohammed, Abdul-Hamid Emwas, Riaz A. Khan

**Affiliations:** 1 Department of Medicinal Chemistry and Pharmacognosy, College of Pharmacy, Qassim University, Buraydah 51452, Saudi Arabia; 2 Department of Pharmacognosy and Medicinal Plants, Faculty of Pharmacy, Al-Azhar University, Cairo 11371, Egypt; 3 Core Labs, King Abdullah University of Science and Technology (KAUST), Thuwal 23955, Saudi Arabia

**Keywords:** halophytes, salt-tolerance, secondary metabolites, phenolics, flavonoids, oxidative stress, immunomodulation, oxidation, anti-cancer activity, cancer management

## Abstract

Halophytes and xerophytes, plants with adequate tolerance to high salinity with strong ability to survive in drought ecosystem, have been recognized for their nutritional and medicinal values owing to their comparatively higher productions of secondary metabolites, primarily the phenolics, and the flavonoids, as compared to the normal vegetation in other climatic regions. Given the consistent increases in desertification around the world, which are associated with increasing salinity, high temperature, and water scarcity, the survival of halophytes due to their secondary metabolic contents has prioritized these plant species, which have now become increasingly important for environmental protection, land reclamation, and food and animal-feed security, with their primary utility in traditional societies as sources of drugs. On the medicinal herbs front, because the fight against cancer is still ongoing, there is an urgent need for development of more efficient, safe, and novel chemotherapeutic agents, than those currently available. The current review describes these plants and their secondary-metabolite-based chemical products as promising candidates for developing newer cancer therapeutics. It further discusses the prophylactic roles of these plants, and their constituents in prevention and management of cancers, through an exploration of their phytochemical and pharmacological properties, with a view on immunomodulation. The important roles of various phenolics and structurally diverse flavonoids as major constituents of the halophytes in suppressing oxidative stress, immunomodulation, and anti-cancer effects are the subject matter of this review and these aspects are outlined in details.

## 1. Introduction

Cancer has long been identified as one of the leading causes of worldwide deaths and is the second leading cause of death in the current millennium; it is still counted as one of the major health problems of the world [1]. Cancer is a heterogeneous disease with complex syndromes and affects different organs [2]. Moreover, cancer is a non-communicable disease that aggressively undergoes abnormal and uncontrolled growth, leading to its spread to other parts of the body, a phenomenon termed metastasis [3]. In the United States alone, about 660,000 cancer deaths, and over 1.75 million new cancer patients, were diagnosed in a single calendar year [1]. Moreover, the American Cancer Society states that one of every three people are at risk of developing cancer in the United States, a rate which reflects the extent of cancer spread around the world [4]. In the Middle East, cancer patients are expected to double from 2018 to 2030 [5]. The factors involved in cancer development are not well understood. However, a list of external and internal parameters has defined cancer-associated factors. External factors, including pollution, radiation, and drug effects [6], have been identified. In addition, the World Health Organization (WHO) includes smoking, alcohol consumption (both light and hard), obesity, and physical inactivity among the primary triggers [7]. The internal factors responsible for cancer triggers, which are unmodifiable and endogenous in nature, are mostly associated with spontaneous mutations during DNA replication, aging, hormonal changes, and inflammation [8,9].

Natural products have long been used in traditional medicine, and their use is still widespread, owing to their availability in various geographic locations around the world. Natural products are still the major source of new drugs, including leads and new drugs alike [10,11,12]. Natural products sourced from plants and animals have been used in prophylaxis, prevention, and treatment of various cancer types [13,14,15]. Dietary vegetables and fruits enriched with phenolics, polyphenols, flavonoids, isoflavones, cucurbitacins, and curcuminoids supplement the human body with essential antioxidants and anti-inflammatory substances that reduce the risk of cancer development [16,17]. Moreover, plant-based natural products have been used in cancer treatments, and more than 3000 plant species have been reported to possess anticancer activity [15,18]. Beginning in the 1950s onwards, thousands of plants have been screened for their cytotoxic and anticancer activity [15,18,19]. The results of these screenings, especially for plants commonly used in traditional medicine, have contributed significantly towards the development of natural anticancer drugs [20,21], and many of these natural products, including *vinca* alkaloids, i.e., vincristine and vinblastine, podophyllotoxins from the *Podophyllum* herb, and paclitaxel from Pacific yew (*Taxus brevifolia*), are used in cancer treatments [15,22,23]. Considering the low specificity, selectivity, and serious side effects of chemotherapeutics, more drug discovery research on novel, effective, specific, and safe anticancer drugs based on natural products are still mandated [24,25]. 

**Figure 1:**
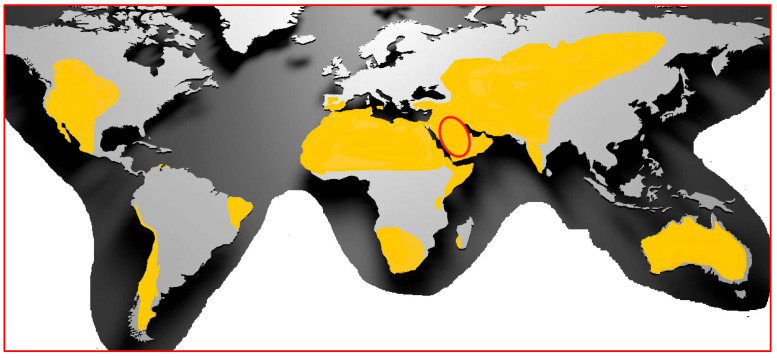
Global arid areas, highlighting regions with increasing salinity and desert progression (yellow). The Kingdom of Saudi Arabia is circled in red.

Plants that grow under the abiotic stresses of high temperature, high salinity, and drought have modulated their physiological bio-mechanistic pathways [26,27,28,29] that protect them from the abiotic factors [30,31]. These pathways may include the up-regulation of genes involved in the oxido-reductive system, osmotic regulation, and metabolite biosynthesis [31,32]. In particular, salinity is an abiotic stress factor that is widespread due to climate change(s) and is considered a major hindrance in agriculture and food security, where salt-stressed irrigated land already exceeds 45 million hectares worldwide [31]. Salinity is a serious problem in Middle Eastern nations, where most of the land may endure this stress by ca. 2050 (Figure 1) [33]. The accumulation of functionally important secondary metabolites is one of the defensive mechanisms used by plants to cope with environmental abiotic stress [34,35]. The accumulation of major secondary metabolites, the phenolics and flavonoids, in salt-stressed plants is mostly related to their over-activation of certain enzymes, such as phenylalanine ammonia lyase and chalcone synthase, which are involved in the biosynthesis of phenolics and flavonoids [34]. These secondary metabolites are also involved in a variety of physiological functions that allow the plant cells to overcome deleterious oxidative stress effects caused by environmental factors [34,35,36,37]. The accumulation and structural diversity of secondary metabolites in stressed plants provide potential therapeutic agents and novel structurally diverse molecular templates, which make them promising compounds for new therapeutic development. The plant metabolites that are produced in response to environmental and other plant defense-related stress factors have shown distinct biological effectiveness and therapeutic potential against communicable and non-communicable diseases, such as microbial infections, oxidative stress, and related disorders, as well as against cancer [38,39]. 

## 2. Salt-Tolerant Plants: Halophytes

**Figure 2:**
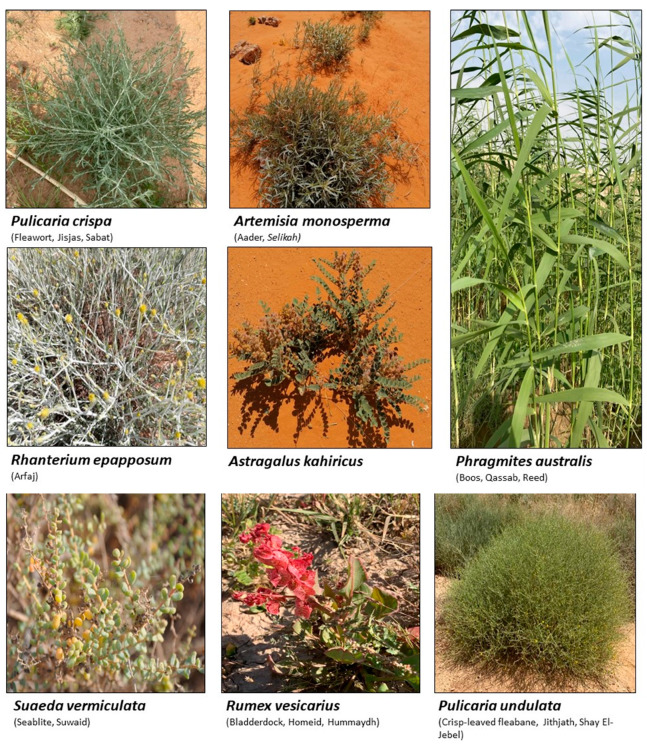
Photographs of some of the halophytes growing in the central region of Saudi Arabia, (magnification ranges between 2× and 3×).

Several types of plants species can efficiently grow and reproduce in high-salinity, dry environments and under drought conditions. These plants have the ability to resist high salinity by modifying their biological and natural properties, including morphologies [40,41,42]. Such plants are scientifically categorized as halophytes, and include more than 1500 species found on all continents of the globe, except Antarctica [43]. The halophytic flora are therefore widely distributed in marshy areas, coastal locations, sandy beaches, and large swaths of deserts with low rainfall, including the mountains across the Arabian desert, plains, and prairies, where the salinity of the soil is comparatively very high in relation to normal tropical and sub-tropical soils [44,45,46,47]. Figure 2 shows some of the halophytic plants growing in the deep desert areas of the central region of the Kingdom of Saudi Arabia. 

Halophytes, which have the ability to overcome the abiotic oxidative stress of high salinity on a more elaborate scale, can do so because of the presence of several antioxidant enzymes and related secondary metabolites produced through the activity of the plants’ inherent enzyme systems, together with the adaptive nature of the plant species; this adaptation is gained through exposure to environmental hardships [48,49,50,51]. The enzymatic defense system includes superoxide dismutase, catalases, ascorbate peroxidases, and glutathione reductase [52]. The enzymatic system plays an elaborate part in various defense mechanisms of these plants which participate through various physiological functions and the biochemical pathways responsible for them. The antioxidant secondary metabolites produced in the halophytes, as a product of the enzymatic activities, act as part of a non-enzymatic defense system [53,54]. This system includes the presence of phenolic compounds of phenylpropanoid nature, such as cinnamic acid derivatives and several C-15 (C6-C3-C6)-skeleton-based flavonoids [55,56,57]. The halophytes accumulate phenolics and flavonoids at higher levels than other plants growing in areas of normal salinity and water conditions [55,58]. However, the presence of phenolic contents in the halophytes depends on the strength of the salinity in their growing environment [59,60]. Halophytes are also a potential source of other secondary metabolites, including alkaloids, saponins, iridoids, sterols, terpenoids, volatile oils, and certain bitter principles [49,61,62,63,64,65,66]. Such phytochemicals, in addition to phenolics and flavonoids, make these plants potential natural sources for newer structural templates, and reservoirs of new and novel molecules sought for the treatment of several diseases [61,67,68]. These molecules, new and known, are also the basis of the claimed bioactivities of these plants. The common and specific pharmacological activities that are claimed in ethnomedicines and other traditional sources are attributed to this diversity of compounds that form the contents of the ingested plants in their extracted, concocted, and other forms, which includes their use as such, and as the whole plant. The halophytes and other plants used in traditional medicines have served populations dwelling in far-reaching areas where modern medicine and its facilities have not penetrated. Halophytes have, for long time, been the crucial component of traditional herbal medicine, and in this capacity, they have also served the nomadic tribes in the Arabian desert [61,67,69,70,71]. Nonetheless, the range of bioactivity of the halophytes covers a broader segment in disease amelioration, and includes plants showing anti-bacterial, anti-fungal, anti-cancer, and anti-viral properties [68,72,73,74]. Halophytes are also used to treat chronic diseases of the liver, heart, and kidneys, including jaundice, hypertension, diabetes, renal insufficiency, and renal calculi, by local and nomadic tribes in various regions where they occur [67,68,72].

## 3. Oxidative Stress and Antioxidants: Halophytes Perspective 

**Figure 3:**
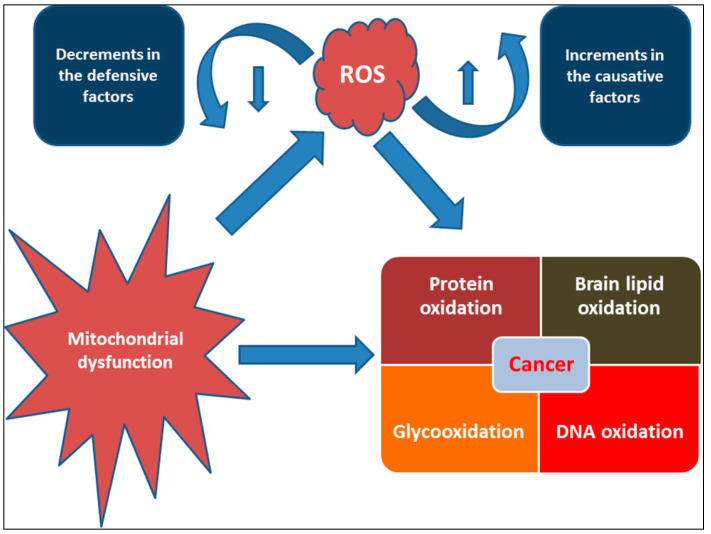
Role of oxidative stress and mitochondrial dysfunctions in cancer development.

**Figure 4:**
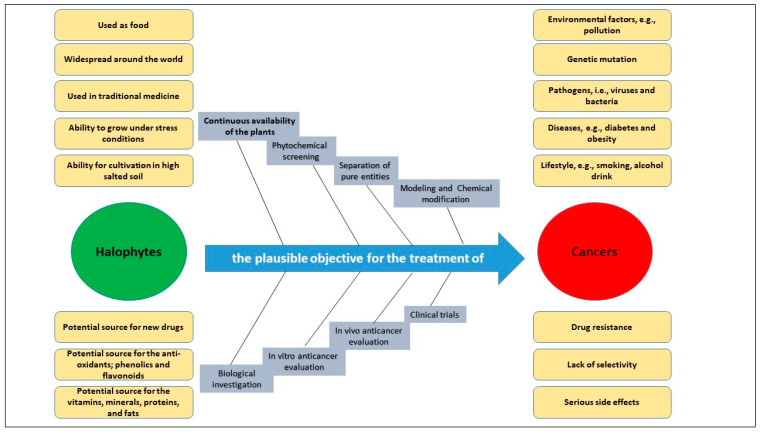
Halophytes as a plausible objective for prospective cancer drugs, and drug leads.

Oxidative stress is a term that indicates the levels of oxidizing agents, e.g., reactive oxygen species (ROS) and reactive nitrogen species (RNS), in the living organism, which at times may be too high owing to the biochemical and physiological factors which cannot be controlled by the internal antioxidant mechanisms of the body to avoid their deleterious effects [75]. The oxidant agents, including ROS, are produced normally as by-products of metabolism [76], and their levels in the human body are maintained by innate biological processes [77,78]. The oxidants are also normally generated as part of the immune response against microorganisms [79]. However, any change in these normal levels due to internal or external factors disturbs the normal functioning of the body, and thus poses health risks in different ways, including risks for and causes of early aging [76,80]. The internal production of ROS primarily takes place in the mitochondrial system through electron transfer processes, also called oxidative phosphorylation, which is responsible for the production of about 90% of the endogenous ROS [81]. However, excessive ROS are produced from dysfunctional mitochondria. The aberrations in oxidative metabolism enzymes, e.g., α-ketoglutarate dehydrogenase complex, pyruvate dehydrogenase complex, and cytochrome oxidase, as well as calcium dyshomeostasis, have been reported to be involved in dysfunctional mitochondria. This dysfunctional status has been linked to the development and progression of several disorders, including cancers [81,82]. Other disease conditions and metabolic disorders have also been linked to oxidative stress, including liver, neurodegenerative, and cardiovascular diseases [76,77,83]. Oxidative stress has well established roles in the initiation, progression, and development of cancers [84,85]. The excessive production of ROS in response to oxidative stress tends to subsequently damage DNA, proteins, and lipids [86] (Figure 3). The growth of tumors related to excessive ROS levels has been found to be associated with the activation of pro-oncogenic signaling pathways, including activation of the mitogen-activated protein kinase (MAPK), JUN N-terminal kinase (JNK), cyclin D1 expression, and extracellular signal-regulated kinase (ERK) pathways, which are all linked to tumor cell activation [87]. It is also reported that ROS concentration is a key factor in augmented tumorigenesis, as well as induced apoptosis. A moderate increase in ROS associated with NF-κB activation, which mediates persistent low-level inflammation, leads to cancer activation [88,89]. Increased cell propagation, metastasis, angiogenesis, and muted apoptosis have all been reported as biological processes that are involved in cancer development, as a response to oxidative stress [90]. Therefore, diets rich in antioxidants, as agents of oxidant-material quenchers, have been suggested to provide protective effects against diseases, particularly cancer(s) [86]. These antioxidant-rich diets, full of phenolics, flavonoids, and carotenoids, are recommended for controlling and treating diseases, including various cancer forms. These contents have also been found to reduce the side effects associated with oncological chemotherapy [87]. The ability of the halophytes to accumulate antioxidants, both the primary and secondary metabolite types, is known to neutralize the intracellular oxidative stress [38,91]. In this context, the widespread distributions of halophytes, their common use in traditional medicine, and their consumption as foods and animal feed, along with their unique ability to survive in harsh environments, have attracted greater interest in several research fields and areas of oncological interventions. The richness of the molecular diversity, especially the ability to produce highly diverse structures and the production of higher quantities of secondary metabolites, including the important phenolics and flavonoids, as compared to normal plants, have attracted continued interest in this segment and the plant species encountered as halophytes [38,39]. This has made halophytes a potential source for anticancer drugs. Figure 4 demonstrates the halophytes as a plausible objective for developing leads, as templates for cancer drug discovery, and in the development of anti-cancer drugs. 

## 4. Roles of Phenolics and Flavonoids in Cancers Preventions and Treatments

Phenolics and flavonoids are known for their potential protective roles against cancer development [92]. In addition, the antioxidant potential of these compounds also has positive health effects on various conditions, including cancer prevention [93]. These products, sourced from different plants, including halophytes, have been reported as free-radical scavengers, transition-metal chelators, and reducing agents for the oxidized forms of metals [92]. The products also have the capacity to inhibit molecular damage and DNA modifications caused by the reactive electrophiles and the intercellular oxidizing agents, which are also associated with mutations and cancer development [94]. Indeed, several research groups have extensively investigated the in vitro anticancer activity of a number of natural phenolics and flavonoids. For instance, coumaric acid, ellagic acid, and kaempferol, the common phenolics in halophytes [95,96], have been reported to inhibit the growth of several cancerous cell lines, e.g., MCF-7 (human breast cancer, breast adenocarcinoma tissue), CAL-27 (human oral adeno-squamous, squamous cell carcinoma of the tongue), HT-29 (human colon cancer, polyposis, small-intestine tissue based), LNCaP (human prostate adenocarcinoma, left supraclavicular lymph node metastasis tissue), and HCT-116 (human colon cancer, adenoma, epithelial tumorigenic colon tissue) [97]. Quercetin and its glycosidal forms are common flavonoids obtained from halophytes [71,98]. Quercetin has been reported to be an apoptosis inducer for several cancers, due to its activation of the TP53 tumor suppressor gene and the deactivation of Bcl-2 gene expression [99]. In addition, quercetin enhances the efficacy and sensitization of the chemotherapeutic agent doxorubicin in MCF-7 cell lines by reducing the multi-drug-resistant protein 1 (MRP1) gene expression levels [100]. Quercetin also induces G1-phase arrest and reduces the expression of Twist, Cyclin-D1, p21, and phospho p38 mitogen-activated protein kinases in MCF-7 and MDA-MB-231 (human, ductal carcinoma, breast cancer late-stage modeled cell lines, triple-negative breast cancer model) [100]. In fact, quercetin has suppressive effects in several cancers, e.g., pancreatic, colon, lung, prostate, gastric, brain, blood, and skin, through different inherent mechanisms, including the cell cycle arrests in different phases. The inhibition of *PI3K*, *MAPK*, *AKT*, *COX-2*, and Bcl-2 signaling, and the activation of *p53*, *Bax*, and *caspase-3*, *-8*, and *-9* have been reported [100]. 

Rhamnetin, chrysin, kaempferol, luteolin, naringenin, hesperidin, myricetin, and apigenin are also common flavonoid aglycones identified in several species of halophytes [101,102,103] that have shown specific anticancer activities [104,105,106]. For example, chrysin inhibits aromatase, an enzyme involved in the biosynthesis of estrogen and a particular target for the treatment of hormone-dependent breast cancer [107]. Chrysin also showed particular anticancer activity against leukemic cell lines and induced apoptosis through activation of caspases and suppression of the Akt signaling pathway [108]. It also has antiproliferative effects against several cancer cell lines, and it has shown potential synergistic effects with certain chemotherapeutic agents, e.g., cisplatin, in HepG2 cells by stabilizing TP53 expression through ERK1/2 activation [109]. Rhamnetin, a 7-methyl quercetin, inhibited the proliferation of MCF-7 and induced apoptosis through caspase-3 and -9 in a dose- and time-dependent manner. It also enhanced the expression of TP53 and microRNA (miR-)34a derived protein levels [110]. Luteolin along with its glycosidal derivative, an abundantly available natural flavonoid in halophytes, has been reported as a potential anticancer agent, and apoptotic inducer against liver, skin, breast, cervical, and brain cancer cell lines [111]. Against breast cancer, luteolin regulated cell proliferation mediated by the epidermal growth factor (EGF). The mechanism involved the lower expressions of p-STAT3 (phosphorylated signal transducer and activator of transcription 3), p-EGFR (phosphorylated epidermal growth factor receptor), p-PKB (phosphorylated protein kinase B), and p-Erk1/2 (phosphorylated extracellular signal-regulated kinase-1/2) proteins in the MCF-7 cell lines [112,113]. In addition, luteolin induced cytoskeleton shrinkage and expression of the epithelial biomarker E-cadherin protein, and it also reduced the mesenchymal biomarkers N-cadherin, snail, and vimentin as part of its apoptotic activity [112]. However, luteolin uses different mechanisms for its anticancer activity in pancreatic cancer cell lines, such as inhibition of EGFR and activation of Bax. For prostate cancer, it was involved in inhibition of fatty acid synthase (FASN); for oral cancer, it activated the cell surface death receptors (FAS); while against lung and gastric cancers, it activated *caspase-3*, *-9*, TP53, and *Bax*; and against cervical cancer, it inhibited PI3K-Akt and activated the death receptor 5 (DR5) [112]. Another flavonoid, naringenin, also works through multiple mechanisms towards eliciting its anticancer effects. Activation of caspase 3 and p38 mitogen-activated protein kinase (p38/MAPK) and suppression of glycogen synthase kinase 3 beta (GSK3β, the key factor in pancreatic cancer progression and invasion) [114,115] have been reported. The downregulation of NF-kB, COX-2, and JAK2/STAT3 have also been reported as part of the naringenin anticancer mechanism [114]. Myricetin stabilized the proliferation of several cancer cell lines, induced apoptosis, and suppressed tumor metastasis [116]. Phenolic acid, such as caffeic acid, has been found present in nearly all halophytes whose anti-cancer activity against different cancers is known [117,118,119]. Caffeic acid reduced the growth of MDA-MB-231 and MCF-7 [119] cell lines and has shown prominent anticancer properties against estrogen receptor positive (ER+) and ER– breast cancer patients [120]. In addition, caffeic acid consumption has shown positive effects against colon and breast cancer cells by decreasing the expression of TRIF, TLR4, and IRAK4 proteins [119]. Other mechanisms related to the anticancer activity of caffeic acid, and other phenolics and flavonoids that are major constituents of halophytes, are summarized in Table 1.

## 5. Traditional Uses of Different Halophytes in Cancers and Cancer-Related Symptoms

Medicinal plants have been used as part of complementary and alternative medicines for different cancer types [141]. More than 3000 plant species are known for their anticancer activity [142,143]. Halophytes, widely distributed in the Arabian desert, have been used in traditional medicine for disease prevention and treatment [68,144]. Certain halophytes have also been used against cancers and in the management of cancer symptoms [145,146,147]. However, published data reporting the ethno-medicinal uses of halophytes in cancer are very limited [72,148]. However, the *Plantago* species (Fleaworts, Plantains), *Rubia cordifolia* (Manjistha, Indian Madder), and *Salicornia herbacea* (Glasswort) are known medicinal halophytes used against cancers [144]. Moreover, the concentrated decoction of *Peganum harmala* (Harmel), *Zizyphus lotus* (Wild jujube), *Asplenium ceterach* L. (Rustyback), and *Calendula arvensis* L. (Field Marigold) have been reported for tumor management [145,146]. Seeds of *Atriplex halimus* (Saltbush) are also used by breast cancer patients in Algeria [147]. Several species from the halophytic genus *Salsola* have been used as a remedy for cancers in Chinese traditional medicine, which includes *Salsola tragus* (Russian-thistle), *Salsola foetida* (Zri-che, Ecchi, Ressal, Aghacel), *Salsola baryosoma*, and *Salsola richteri* (Kata-kara, Cherkez) [149]. The common use of these plants to manage and treat cancers in traditional medicines is an incentive for researchers to further investigate their constituents and examine their effects on various cancer cell lines, as well as their effects in vivo. 

## 6. Evaluating Anti-Cancer Activities of Halophytic Plants Extracts 

A considerable number of reports are available in which halophytic plant extracts and their fractions have been screened for cytotoxic and antiproliferative effects [150,151,152,153,154]. Most of these reports evaluated the anticancer activity of plant extracts and fractions that were identified by liquid chromatography–mass spectrometry (LC–MS) and gas chromatography–mass spectrometry (GC–MS) [153,154,155] for the presence of the chemical constituents. Nuclear magnetic resonance (NMR) spectroscopy has also been used for identification and quantification of the chemical constituents and their compositions in the mixture [156]. Quantitative spectrophotometric analysis has also been conducted [154,157]. For instance, *n*-hexane, dichloromethane, methanol, and water extracts from the xero-halophytic species *Reaumuria vermiculata* have been phytochemically investigated and evaluated for their anticancer activity together with their anti-inflammatory and antioxidant effects [158]. The *n*-hexane and dichloromethane extracts had the highest cytotoxicity against A-549 lung carcinoma cells (IC_50_ values of 17 and 23 µg/mL, respectively), while the methanol extract of the plant showed the highest quantities of phenolics and flavonoids [158], indicating that the non-polar constituents were responsible for the plant’s anticancer properties. Some studies also demonstrated the anticancer activity of whole-plant extracts. Mohammed, et al. [154] reported the anticancer activity of an aqueous alcoholic extract from *Pulicaria undulata* growing in Saudi Arabia. They found that the plant extract had a potent cytotoxic effect against several cancer cell lines, including MCF-7, K562, and PANC-1, with IC_50_ values ranging from 519 to 1535 µg/mL. The extract also inhibited normal fibroblast cell growth at IC_50_ values greater than 4000 µg/mL, indicating the high selectivity index for the cancer cell lines. According to the authors’ conclusions, the accumulation of polyphenols and flavonoids in the plant extract was responsible for the plant’s anticancer effects [154]. In addition, the anticancer activities of essential oil constituents of some of the halophytic plants have also been reported. For example, oils obtained from *Mentha piperita* growing in the Experimental Halophytes Growing Base at the Shandong Academy of Sciences, Jinan, China, exhibited cytotoxic activity in pulmonary carcinoma (SPC-A1, human, lung cancer), K562 (human, chronic myelogenous leukemia), and gastric cancer (SGC-7901, human, first isolated from surgically resected metastatic lymph node) cell lines with IC_50_ values which ranged from 10.89 to 38.76 µg/mL [159]. In vitro and in vivo assays were used to investigate the chemopreventive impacts of halophytes. Six halophytes were also examined in vitro for their stimulation of NAD(P)H: quinone oxidoreductase-1 (NQO-1) in the hepatoma cells (Hepa-1c1c7) murine culture. The results revealed that *Ferocactus herrerae*, *Aptenia cordifolia*, *Carpobrotus edulis*, and *Ferocactus glaucescens* were the most active chemopreventive plants [160]. *Tamarix gallica* methanolic extract was demonstrated in vivo to have chemopreventive activity against liver cancers, induced by diethylnitrosamine and 2-acetylaminofluorene, which worked through restoring the detoxifying cellular antioxidant enzyme ornithine decarboxylase and DNA synthesis [161]. Several investigations on other halophytes have been conducted in similar fashion with the purpose of identifying the plant components and quantifying the presence of key secondary metabolites and their cytotoxic activity (Table 2).

The isolation, purification, and characterization of pure secondary metabolic ingredients from plant extracts involve time-consuming, technically advanced and difficult work in chromatographic and spectroscopic processes that lead to structure elucidations of the isolated pure compounds. Furthermore, the isolation techniques may not provide sufficient quantities of pure chemicals for undertaking in vivo and in vitro biological and pharmacological evaluations. Computer-assisted in silico receptor binding has paved the way towards a better understanding of the binding process and its requirements in energy and geometry of the ligand and the host protein through which the chosen compound exerts its biological functions [187,188]. In silico applications together with LC–MS and/or GC–MS analyses have revealed the particular protein involved in cancer development and activation of the substrate responsible for the cancer onset. The technique has been used to investigate the anticancer mechanisms of various plant-extract-based components [189,190]. In vitro and in silico experiments on the anticancer activities of particular enzymes from halophytic plant-extract compounds are reviewed [153,191,192]. The cytotoxic effects of *Zygophyllum coccineum* aqueous ethanolic extract on three cancer cell lines, MCF-7, HCT-116, and HepG2, have been explored. An in vitro suppression of human topoisomerase-II enzyme, and the in silico receptor-site binding by major compounds of *Z. coccineum* are also reported [153]. The results showed that the *Z. coccineum* extracts may have potential anticancer activity, as supported by the higher inhibition scores of the major constituents of the plant against human topoisomerase-II (IC_50_ value 45.05 ng/mL) in comparison to the standard enzyme inhibitor staurosporine (IC_50_ value 135.33 ng/mL). The binding energy requirements, obtained through in silico experiments, substantiated the findings [153]. Another example of in silico predictions involving the methanol extract compounds of *Moricandia sinaica* shoot have shown potential for cytotoxic activity, thereby corroborating the approach. In parallel, a GC–MS analysis identified 2-tridecen-1-ol as the major component in *M. sinaica* methanolic extract for higher binding energy of M-phase inducer phosphatase 2 (CDC 25 B) in comparison to the M-phase inducer phosphatase 1 (CDC 25A), which also suggested a plausible molecular mechanism for the extract’s anticancer effects [193]. Recent studies also demonstrated that *Suaeda vermiculata* extracts exhibit anticancer activity in HepG2 and HepG-2/ADR resistant cell lines. The plant extracts also improved the sensitivity of the HepG-2/ADR cells to doxorubicin, a known anti-cancer compound, which was evidenced by the in vitro MTT assay using combinations of the extracts with doxorubicin. The in silico binding affinity for the three ATP-binding cassette proteins responsible for the efflux of chemotherapeutic agents also indicated in this direction [194].

## 7. Isolated-Purified Anti-Cancer Agents from Different Halophytes

**Figure 5:**
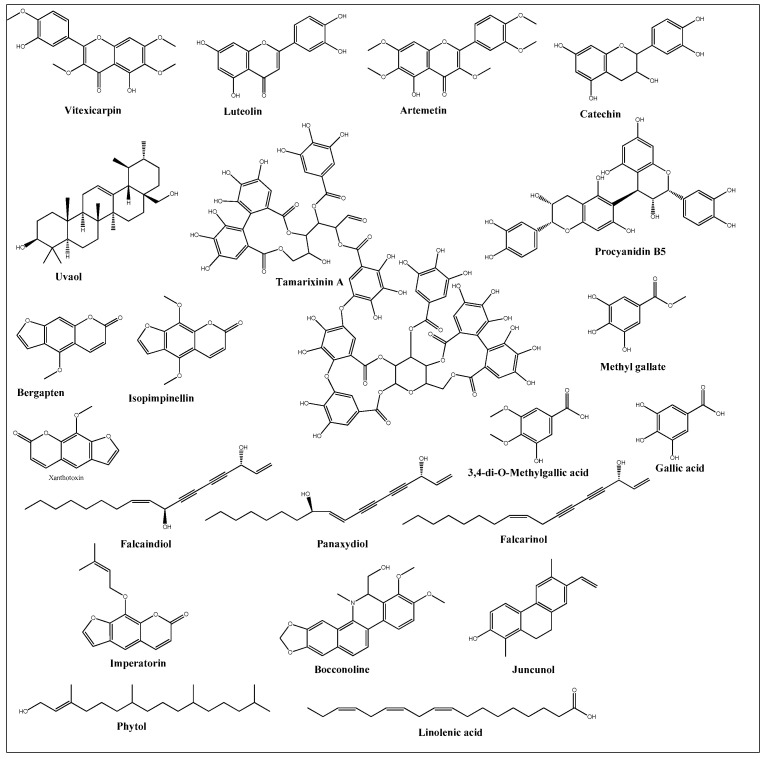
Chemical structures of pure compounds isolated from different halophyte plants with reported cytotoxic activity.

Anticancer activities as anti-proliferative and cytotoxicity of halophytic plant extracts have been widely studied; nonetheless, the anticancer activity of pure chemical compounds isolated, purified, and characterized from halophytes (Figure 5) is less encountered. Flavonoids, the most extensively distributed class of compounds in halophytes, have also been evaluated for their antitumor activity. For example, luteolin, vitexicarpin, and artemetin have been isolated from the halophyte *Vitex rotundifolia* and tested for their anticancer activity [195]. Among the three compounds, vitexicarpin was the most active cytotoxic agent against human gastric adenocarcinoma (AGS) and human colon cancer HT-29 cell lines, with IC_50_ values of 6.9 and 22.8 µM, respectively. Vitexicarpin also induced apoptosis by upregulating the expression of TP53 and p21 and downregulating the expression of Bcl-2 at a concentration of 25 µM [195]. Catechin, epicatechin, and procyanidin B5 were isolated from *Carpobrotus edulis* and shown to possess cytotoxic activity against MDR1-transfected mouse lymphoma (L5178) cells, with IC_50_ values equal to 12, 6, and 13 mg/L of the pure compounds, respectively [166]. In addition, three triterpenoids and a monogalactosyl diacylglycerol compound isolated from the same plant demonstrated antiproliferative potential in MDR1-transfected L5178 cells. In addition, uvaol (triterpene) exhibited the highest relative fluorescence factor (FAR) value and considerable inhibition of P-glycoprotein [166]. Halophytes are also a rich source of phenolic acids since they abundantly produce phenolics as a defensive mechanism against the oxidative stress of salinity. Therefore, phenolics have been represented in all reported phytochemical analyses of halophytes. In addition, some reports have investigated the isolation and anticancer effects of halophyte phenolics. For example, several phenolics have been isolated from *Tamarix nilotica*, including stamarixinin A (ellagitannin), gallic acid, methyl gallate, and 3,4-di-O-methylgallic acid, and these products have been examined for their antiproliferative activity against lung adenocarcinoma cell lines A549 [196]. Among these four compounds, gallic acid exhibited the highest cytotoxic effect, with an IC_50_ value of 10.5 µg/mL. Other classes of natural products have also been identified from halophytes and found to exhibit anticancer activity. For instance, bitter principles of the furanocoumarin-type, i.e., bergapten, isopimpinellin, xanthotoxin, and imperatorin, as well as polyacetylene alcohols, i.e., panaxydiol, falcaindiol, and falcarinol, were isolated from *Glehnia littoralis*, a halophytic species, and were shown to have dose-dependent antiproliferative effects against HT-29 cell lines. Among all of the isolated compounds, falcaindiol was the most active cytotoxic agent against HT-29, with an IC_50_ of 35 µM [174]. The pure alkaloid bocconoline, isolated from *Glaucium flavum*, demonstrated strong cytotoxic effects against MDA-MB-231 cell lines, with an IC_50_ of 7.8 µM [197]. Juncunol (7-vinyl-9,10-dihydro-1,6-dimethylphenanthren-2-ol) was isolated from *Juncus acutus* ether extract, and exhibited potential cytotoxic activity against HepG2, HeLa, and MDA-MB-468, with IC_50_ values ranging from 13 to 20 µM together with a highly selective index compared to its effect on the normal cell lines mTEC and S17 [198]. Finally, *Salicornia herbacea* polysaccharide inhibited cell growth and induced apoptosis in HT-29 cell lines [181]. The mechanisms by which these polysaccharides exhibit their anticancer activity has been attributed to their effectiveness in inducing G2/M cell cycle arrest at a dose of 4 mg/mL and the inhibition of cyclin B1 and Cdc2 *mRNA*, which leads to inhibition of HT-29 cell-line proliferation [181]. 

## 8. New Anticancer Agents from Halophytes

**Figure 6:**
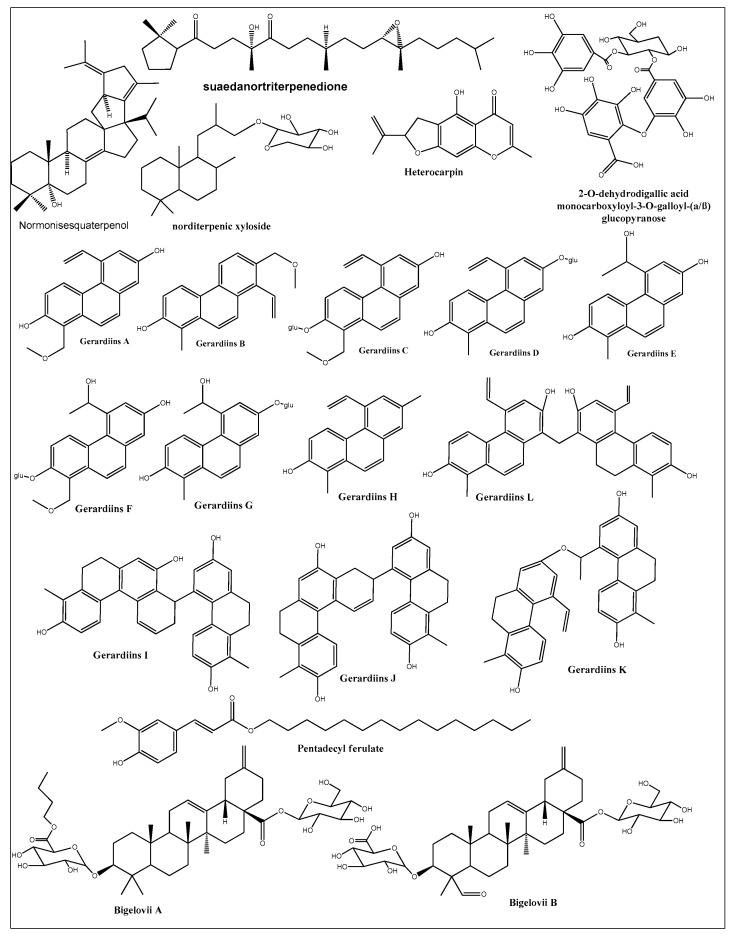
New and first-time reported cytotoxic compounds isolated from different halophytic plant species.

A number of new and first-time isolated cytotoxic compounds from different halophytic plants are represented in Figure 6. The list includes phenolics, flavonoids, coumarins, saponins, and alkaloids. Some of these new compounds have shown very promising anticancer activity. Heterocarpin, a furochromone obtained from *Corydalis heterocarpa*, suppressed the growth of AGS, HT-29, HT-1080, and MCF-7 cell lines [199]. The compound also induced apoptosis in AGS cells by regulating the Bax-Bcl-2 ratio, overproducing caspases, and suppressing the X-linked inhibitor of apoptosis protein (XIAP). Heterocarpin also induced inhibition of NFκB and activated JNK and ERK pathways in AGS cells. Another study [200] reported 12 new phenanthrene-based gerardiins (gerardiins A–L), which were isolated for the first time from *Juncus gerardii* methanol extract and were found to be anti-cancerous in nature. Other phenanthrenes and flavonoids, such as apigenin and luteolin, were also isolated for the first time from *J. gerardii*, and MTT assays of these compounds against 4T1 and MDA-MB-231 confirmed their cytotoxic activity, with IC_50_ values ranging from 5.6 to 8.0 µM. Pentadecyl ferulate, isolated from *Salicornia herbacea* [201], exhibited cytotoxicity against HepG2 and A549 cell lines at EC_50_ values of 56 and 48 µM, respectively. Several known compounds of fatty acids, long-chain alcoholic hydrocarbons, phthalates, steroids, and phenolics were also isolated from *Salicornia herbacea* acetone extract [201]. Among these, phytol selectively inhibited HepG2 growth at an EC_50_ dose of 78 µM, while linolenic acid inhibited the growth of HepG2 and A549 cell lines at EC_50_ values of 65.35 and 83.23 µM, respectively. However, all other compounds were inactive until they reached dose levels of 200 µM. Two additional saponins, bigelovii A and bigelovii B, were discovered from another *Salicornia* species, *Salicornia bigelovii* [202]. Bigelovii A inhibited the growth of HL-60, MCF-7, and HepG2 cell lines, with IC_50_ values of 6.18, 78.08, and 13.64 μM, respectively. Bigelovii A also exhibited strong cytotoxic effects against several breast cancer cell lines (MCF-7, MDA-MB-231, and MDA-MB-468), and induced apoptosis by downregulating the NF-kB signaling genes Cyclin D1 and COX-2 [203]. Known compounds of 30-nortriterpenoid and oleanane-type triterpenoid glycosides were also isolated from *Salicornia bigelovii*, but only the 3-*O*-β-*D*-glucuropyranosyl-30-norolean-12,20-(29)-dien-28-oic-acid-28-*O*-β-d-glucopyranoside was cytotoxic-active against HL-60 cell lines, with an IC_50_ value of 31.87 μM [202]. Moreover, six other compounds, namely normonisesquaterpenol, suaedanortriterpenedione, aromatic monoterpenic ester, norditerpenic xyloside, alkylated β-naphthol, and β-methoxy naphthalene, were isolated, for the first time, from *Suaeda monoica* and tested for their potential cytotoxic and apoptotic activities [129]. Among these, suaedanortriterpenedione, normonisesquaterpenol, and norditerpenic xyloside showed the highest cytotoxic activity and apoptotic effects by downregulating caspase-3 and -7 [204]. Two new gallic-acid-based tannin derivatives were isolated from *Reaumuria vermiculata* aqueous methanolic extract and were identified as 2-*O*-dehydrodigallic acid monocarboxyloyl-3-*O*-galloyl-(α/β)-glucose, and vermiculatin. Both compounds showed antiproliferative activity against the prostate cancer cell line PC-3, with IC_50_ values at 1.5 and 0.45 M dose levels, respectively [180]. The isolation of these new and structurally novel compounds from halophytic plants suggests that the halophytes are promising candidates for the discovery of new and structurally novel anticancer and chemoprotective agents. 

## 9. Immune System, Immunity, and Cancer Immunotherapy: The Flavonoids and Polyphenols Perspective

The immune system protects a living being against diseases, and its participation and functional variability can be promoted and enhanced by certain substances that are referred to as immunomodulators. The use of a synthetic immunomodulator, which produces a quick disease response but often leads to side effects such as nausea, bone marrow degradation, and low red (thrombocytopenic purpura) or white (agranulocytosis) blood counts. 

Pro-active immune systems maintain regular immunological and biological functions in best order and provide protection against unwanted pathogens, infection, and diseases. The immune system helps to elude hypersensitivity reactions and immune-related disorders, including severe immune diseases. The immunomodulation in its functional entirety encompasses activities towards alterations in immune response(s), which comprises response-induction, manifestation, magnification, and inhibitory action in parts and/or stages in the immune response towards enhancements and/or reductions in the immunological set-up’s sensitivity to also yield decreases in response(s) to match the biosystem’s requirements. Phytochemicals, e.g., phenolic compounds, flavonoids, anthocyanins, tannins, alkaloids, lectins, glycosides, saponins, terpenoids, sterols, and polysaccharides, are a source of natural immunomodulators, and also contribute roles in cancer prevention and probably a cure [205]. 

Different immunomodulators affect different constituents of the set-up of the immune system. These immunomodulators may broadly respond to all immune threats, or function against certain types of pathogens or threats only by certain immunomodulators that target specific pathways to exert their responses. Small molecular weight (SMW) entities, polymerics, e.g., tannins and polysaccharide, biomolecular entities including protein-based compounds, as well as antibodies, more specifically monoclonal antibodies function as immunomodulators in two broad ways to either suppress the over-rated response to the immune-threat at site(s), or enhance the immune system’s reactivity to control the situation. The immunosuppressant contributes to control autoimmune disorders (immune system mistakenly attacking healthy tissue), while the immune enhancers or the commonly termed immunomodulators, help contain cancers. The immunomodulators are also useful in various other diseases, i.e., infections, rheumatoid arthritis (RA), multiple sclerosis (MS), psoriasis, psoriatic arthritis, Sjögren’s disease, lupus, inflammatory bowel disease (IBD), ulcerative colitis, Crohn’s disease, and several allergic conditions, including common cold/food/environmental allergies, asthma, and eczema.

Cancer immunotherapy is managed by turning off signals through immune checkpoint inhibitors so that immune cells stop responding to cancer cells, also by the functions of the cytokines involved in immune system signaling, and through chimeric antigen receptor (CAR) T-cell therapy in which immune T cells are procured from blood and are in vitro modified to respond to the cancer, also through cancer vaccines (e.g., melanoma and prostate cancer vaccine), and synthetic drugs, e.g., thalidomide and lenalidomide, to target immune pathways, and corticosteroids. The role of traditional medicine, especially the flavonoids, fits in this category, which, depending upon the formulation and user conditions, can be administered orally, intravenously, intramuscularly, and also topically, especially the corticosteroids, for skin cancers. However, the immunomodulators lead to weakening of the natural status of the immune system, and generate fatigue, nausea, diarrhea, headaches, body aches, pains, site swelling, and redness, and medication after-effects include allergic reaction(s).

Flavonoid- and polyphenol-rich extracts/fractions with anti-cancer activity from halophyte plants have also been discovered (Table 2), and the anti-cancer activity of certain individual flavonoids and polyphenols, also sourced from non-halophyte origins, has been confirmed (Table 1). Several plants, their constituents, and extracts are known to produce immunomodulatory activity as part of the anti-cancer dose regimens. The immunomodulatory actions of flavonoids and other constituents in honey are well known, and the use of honey as an adjuvant in cancer therapy has been recommended [206]. The effects of three flavonoids, namely apigenin, luteolin, and quercetin, on NK cell activity against lung cancer cells, and on the secretions of the cytotoxic granules perforin and granulysin were also examined [207]. Naringenin, a flavonoid product, is known to modulate mouse J774 macrophages upon infection with *C. trachomatis*, through modulation of TLR2, TLR4, and CD86 receptors and down-streaming of the MAPK (p38) pathway [208]. Silymarin [209], and cocoa [210] have also been indicated in apoptotic immunomodulation, with uncertain mechanisms of action for the latter. 

The aqueous extract of *Moringa oleifera* leaves [211] and flavonoids of *Phyllanthus niruri* [212] have been observed to work as immunomodulators, and *M. oleifera* has been recommended based on detailed investigation of its anti-cancer activity. Flavonoid–metal complexes as promising anticancer metallo-drugs have been proposed, which require further mechanistic investigations with regard to the immunomodulation and its involved pathway [213]. The Natural Medicines Comprehensive Database (NMCD) and the Lawrence Review of Natural Products–Monograph System has provided referenced and evidence-based studies on immunomodulatory natural-product-based sources [214].

## 10. Molecular Basis of Immunology, and Major Cancer Immunotherapy Mechanisms: The Flavonoids and Polyphenols Stand-Point 

Flavonoids as part of different chemical structures, e.g., flavanone, flavone, flavonol, catechin, isoflavone, chalcone, and anthocyanin also enhanced the activities of several enzymes [215]. Recent studies have shown that they possess immunomodulatory properties as well. However, most investigations were performed in vitro; only a handful of studies are available in animal models, and very random human studies are reported. The in vitro studies are on single flavonoids, mostly aglycones, and at supra-physiological (physiologically non-attainable in biosystems) concentrations. Certainly, more animal-model-based studies, studies involving humans, and epidemiological studies with prospective randomized trials are needed to unravel the immunomodulation effects of the individual flavonoids. Further details on enzyme functions, regulation of the involved genes, and protein expressions are invaluable towards understanding the phenomenon, although a certain number of animal studies and epidemiological and human intervention trials have been reported [216]. Flavonoids exert specific immunomodulatory effects that are crucial to control and treat different types of cancers. Immune cells, T-lymphocytes, play an important role in protecting the immune system and pathogenesis of certain diseases. Among important mediators of the immune system, mTOR is crucial in T-lymphocytes. The impact of flavonoids on the mTOR pathway is well-known, as they suppress mTOR activity and subsequently induce T regulatory subsets for the immune response and immune-related activity [217].

Thus, the immune system, which plays a critical role in cancer prevention, contributes through innate and acquired immunity. Innate immunity lacks specific recognition and responds to all pathogenic disturbances regardless of their nature [217], while acquired immunity recognizes pathogens and responds accordingly. The innate part consists of immune and non-immune components, whereas acquired immunity works only through immune elements. The prime functions of the acquired immune set-up rely on immune cells, i.e., B and T-lymphocyte cells, which help to recognize pathogens according to their antigen receptors. The B cells produce antibodies to neutralize pathogenic activity, and consequently opsonize pathogens for phagocytosis. Of the T-cells, the T cytotoxic cells (T CD8+) kill cancer cells, and the T helper cells (T CD4+) secrete a wide range of cytokines and other mediators to involve B lymphocytes and macrophages [218,219,220]. Cytokines produced by the Th (T helper) cells direct the antibodies produced by B cells and activate the monocytes and macrophages [221].

The effect of flavonoids on certain inflammatory cytokines, i.e., TNF-α [222,223,224], which plays an important role in mediation of cancers, is affected by flavonoids exerting specific effects on the PI3K/Akt/mTOR signaling axis (phosphatidylinositide-3-kinases/protein kinase, mammalian target) in the cancer cells. The cancer cells’ proliferation, which is known to be affected by different structure-based flavonoid molecules, together with the metabolic similarities between the cancer cells and the activated Th cells, indicate the relationships between the flavonoids, Th cells, TNF- α, and the PI3K/Akt/mTOR signaling axis. In this context, certain flavonoids significantly increased mTOR suppression and induced apoptosis in renal cancer cells [225], by arresting the melanoma cells at G2/M phases, inducing autophagy. In vitro studies have confirmed the inhibitions of Akt-, mTOR-, and P70S6K-related activities of the flavonoids and certain polyphenolic compounds in breast and prostate cancer cell lines [226]. Compounds like curcumin are known to inhibit Akt and mTOR functions in the presence of EGF, a ligand for the EGF receptor. The Akt/PI3K/mTOR axis, which is among the vital downstream signaling pathways in cancer promotion, may be a promising find towards cancer therapy, even after EGFR activation [227], through involvement of a number of natural flavonoids and polyphenolic compounds, including curcumin [228]. Other flavonoids include fisetin and quercetin. Some flavonoids also activated the AMPK and PTEN in non-small lung cancer cells [229], wherein the AMPK and mTOR played contrary roles in metabolism, while the mTOR-, PI3K- [229], and Akt-related functional activities were observed in prostate cancer cell lines [230]. The AMPK activation and mTOR suppression resulted in both survival and proliferation failures of the cancer cells [231]. In lymphoma cells, quercetin also inhibited Wnt/catenin pathways, together with the Akt/PI3K/mTOR pathways [232,233]. In a cervical cancer cell line, cell cycle G2/M, arrest and release of cytochrome-C, an apoptotic indicator, was also observed [234]. Certain pomegranate polyphenols also reduced the IGF expression in the colon cancer cell lines, together with suppressing the Akt and mTOR expressions [235]. Moreover, the aryl hydrocarbon receptor (AhR) pathway was also found to be active in certain cancer cells together with the Th cells [236]. In prostate tumor cells, the AhR exhibited abnormal expression, deletion, and/or inhibition, which resulted in inhibition of tumor growth. Through AhR suppression, G0/G1 cell-cycle arrest, and apoptosis, anti-cancer action of quercetin was observed in a prostate cancer cell line [236]. However, the exact mechanism of the AhR involvement with the quercetin anti-cancer action is still unknown. AhR has been proposed to activate the Akt/PI3K/mTOR axis, and AhR inhibition is thought to lower the PI3K functions and restore sensitivity to apoptosis, as observed in mouse-model hepatoma cell lines [237]. Owing to the metabolism’s similarities between the activated Th cells and the cancer cells, the involved polyphenols also suppressed the mTOR activity in the Th cells; in this way, the differentiated regulatory T cells also suppress unwanted immune responses against the self-antigens and work towards anti-cancer activity. 

Virgin Th cells are initially catabolic in nature, but turn anabolic after their differentiation and activation. The unactivated, catabolic Th cells are unable to respond to pathogens, and the PI3k/Akt/mTOR pathway only gets up-regulated upon the activation. The accumulation of certain metabolites is thought to induce Th differentiation and their activation. The individual flavonoids’ exactly defined molecular mechanisms are not well understood. However, both the Th and the cancer cells use glycolysis for energy, and the PI3k/Akt/mTOR axis is suppressed. The Th cells’ effector differentiation is reduced, and T regulatory cells are induced for immunomodulation purposes.

Among other mechanistic involvements, the polyphenols, as known, regulate immunity by interfering with the immune cell modulation, pro-inflammatory cytokine synthesis, and gene-expression controls. These compounds inactivate the NF-κB (nuclear factor kappa-light-chain-enhancer of activated B cells), and control the MAPK (mitogen-activated protein kinase), as well as arachidonic acid pathways. In addition to involvements of PI3K/Akt/mTOR, the inhibition of IKK/JNK (kappa kinase/c-Jun amino-terminal kinases) and the JAK/STAT pathways are also involved. These products also suppress TLR (toll-like receptor) and suppress the pro-inflammatory gene expressions. 

The anti-oxidant activity and inhibition of certain enzymes also contribute to this effect. Enzymes involved in ROS production, i.e., xanthine oxidase and NOX (NADPH oxidase) are inhibited, while anti-oxidant enzymes, i.e., SOD (superoxide dismutase), CAT (catalase), GSH (glutathione), and Px (peroxidase), are upregulated. Moreover, the compounds are also known to inhibit PLA2 (phospholipase A2), COX (cyclooxygenase), and LOX (lipoxygenase), which leads to a reduction in PGs (prostaglandins) and LTs (leukotrienes), as well as production of inflammation antagonism. Thus, polyphenols and flavonoids play an important role in prevention and progression of various diseases, including cancers [238].

## 11. Summary and Future Prospects

Halophytes are a group of plants belonging to different families with a unique ability to survive and reproduce in high salinity, marshes, and drought ecosystems. They have a distinctive ability to biosynthesize and accumulate various secondary metabolites, including phenolic acids, flavonoids, polyphenols, alkaloids, bitter principles, volatile oils, and saponins. These metabolic products are the components responsible for exhibiting various biological and pharmacological activities, the prime reasons for which the halophytes are used in traditional medicine. In their distribution areas, especially in dry and desert climatic regions, halophytes are often used as foods, e.g., in salads; as animal feed; for the treatment of various ailments; and in other common uses with economic and technical benefits. Halophytic plants grow throughout the planet. As advancing desertification is predicted to accelerate their spread, halophytic species are expected to become distributed in newer geographic areas. Considering the anticancer potential of many of the compounds abundant in halophytes, there is an inevitable and renewable source for drugs and drug leads against different cancers. This review has provided examples of halophytes and their constituents used in the treatment of cancer and its various symptoms in folk medicine. The effectiveness of these plants, their parts, extracts, and fractions in ameliorating the cancer types, together with the immunomodulatory activities of plant extracts and the pure isolated products from these plants, are discussed. The discovery of new molecular templates, leads for further development, and new and structurally novel compounds with activity against various cancer cell lines are also detailed. The structural modifications, SAR-based approaches, and new product leads and potent anti-cancer compounds, together with plant components in mixture as part of extracts and sub-extracts/fractions, have great potential in drug discovery, drug development, and complementary and alternative medicine as part of mono- and combined therapies, to change the paradigm in cancer chemotherapy and the field of oncology at large. The potential prophylactic, therapeutic, and economic benefits should gain the attention of governmental bodies, agricultural entrepreneurs, bioengineers, medicinal researchers, and pharmaceutical manufacturers in taking the lead in this field.

## Figures and Tables

**Table 1:** Common phenolics and flavonoids found in halophytes, and their major anti-cancer(s) mechanism.

Sr.	Compound	Structure	Mechanism(s) of Action	References
1.	Luteolin	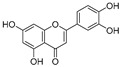	Regulates cell proliferation mediated by EGF, leading to the suppression of p-STAT3, p-EGFR, p-PKB, and p-Erk1/2 expression in MCF-7; induces cytoskeleton shrinkage and the expression of E-cadherin, while reducing the expressions of mesenchymal biomarkers N-cadherin, snail, and vimentin; inhibits EGFR and activation of Bax in pancreatic cancer; inhibits FASN in prostate cancer; activates FAS in oral cancer, and caspase-3, -9, TP53, and Bax in lung, gastric, and liver cancers; inhibits PI3K-Akt and DR5 in cervical cancer.	[112,113]
2.	Quercetin	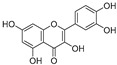	Activates TP53 gene, inhibits Bcl-2 expression; enhances efficacy and sensitization of doxorubicin against MCF-7 by reducing multidrug-resistant protein 1 gene expression level; induces G1-phase arrest and reduces the expression of Twist, Cyclin-D1, p21, and phospho p38 mitogen-activated protein kinases in MCF-7 and MDA-MB-231; inhibits PI3K, MAPK, AKT, COX-2, and Bcl-2 signaling and activates TP53, Bax, caspase-3, -8, and -9.	[99,100]
3.	Chrysin	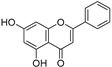	Inhibits aromatase enzyme in hormone-dependent breast cancer; induces apoptosis by caspase activation, Akt suppression, and stabilization of TP53 expression through ERK1/2 activation.	[107,108,109]
4.	Rhamnetin	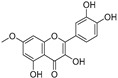	Upregulates caspase-3 and -9 and enhances TP53 protein and microRNA (miR-)34a.	[110]
5.	Myricetin	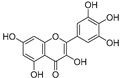	Arrests G-quadruplex structure, represses hTERT expression in MCF-7; attenuates neoplastic transformation of cancer cells; suppresses cyclin-dependent kinase 1 (CDK1), PAK1, MEK, phosphorylated (p)-ERK1/2, β-catenin, cyclin D1, PCNA, and survivin.	[121,122]
6.	Hesperetin	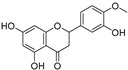	Reduces cellular proliferation by reducing cellular glucose uptake in MDA-MB-231; suppresses insulin receptor-beta subunit (IR-beta) and Akt; inhibits angiogenesis and metastasis by suppressing COX-2, MMP-2, and MMP-9; activates caspases and suppresses Bcl-2 and Bax to induce apoptosis.	[123,124]
7.	Apigenin	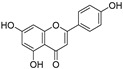	Induces cell cycle arrest, apoptosis, and autophagy and suppresses cancer migration and invasion by inhibiting NEDD9/Src/AKT, PI3K/AKT, ERK1/2, JAK/STAT, Wnt/β-catenin, SAPK/JNK, and FAK molecular pathways and upregulating ATM/ATR and AMPK expression; suppresses overexpression of ERβ in breast and prostate cancerous cells.	[125,126]
8.	Kaempferol	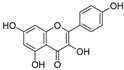	Upregulates TRAIL receptors and induces apoptosis in SW480 (human colon cancer line); induces apoptotic genes TP53, PUMA, NOXA, Bax, BCL-2, Apaf-1, caspase-9, and cytochrome c; downregulates Bcl-2 and Bcl-xL expression; reduces iNOS, COX2, CRP, and NF-κB protein levels; inhibits cancer invasion of breast cancer by blocking the PKCδ/MAPK/AP-1 cascade and subsequent MMP-9 expression.	[127,128,129]
9.	Coumaric acid	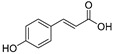	Reduces the expression of COX-2, IL-6, TNF-α and PGE2; downregulates EGFR and GRP78 and activates the unfolded protein response (UPR) leading to apoptosis in cancer cells; modulates the expression of *microRNA*s in gastric cancer cells; induces Nrf2 transcription factor in colon cancer.	[130,131,132,133]
10.	Ellagic acid	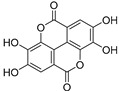	Reduces P-VEGFR2, MAPK, and PI3K/Akt expression in MDA-MB-231, descreases Bcl-2 levels, increases Bax levels, and inhibits SphK1 and integrin-linked kinase (ILK); reduces eicosanoid synthesis and downregulates the heme oxygenase (HO) system in prostate cancer.	[130,131,132,133]
11.	Caffeic acid	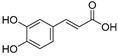	Decreases the expression of TRIF, TLR4, and IRAK4 proteins in breast cancer cells; enhances HO-1, GCLC, and GCLM expression by the Nrf2/ERK pathway in liver cancer cells; enhances the activity of caspases and p53 enzymes and blocks Bcl-2 activity in cervical cancer.	[119]
12.	Ferulic acid	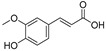	Stops cell division by upregulating the expressions of *ATR*, *ATM*, *CDKN1A*, *CDKN1B*, *E2F4*, *RB1*, and TP53 and downregulating the expressions of *CCND1*, *CCND2*, *CCND3*, *CDK2*, *CDK4*, and *CDK6* in PC-3 cells; upregulates the expressions of *CASP1*, *CASP2*, *CASP8*, *CYCS*, *FAS*, *FASLG*, and *TRADD* and downregulates the expressions of BCL2 and XIAP in LNCaP cells; inhibits autophagy-related proteins such as LC3-II, Beclin1, and Atg12-Atg5.	[134,135]
13.	Cinnamic acid	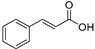	Regulates oncogenes c-myc, c-fos, and *TP53*.	[136]
14.	Epicatechin (R=R1= H)/epigallocatechin (R=OH, R1=H)/epigallocatechin-3-gallate (R=OH, R1=gallic acid)	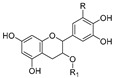	Enhances the gene expression of GST, TP53, PTEN, CYP450, and Bax; suppresses VEGF, COX-2, and NF-KB activity.	[137]
15.	Chlorogenic acid	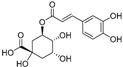	Reduces the inflammatory markers IL-1β, IL-6, IL-8, COX-2, and TNF-α by down-regulating NF-KB expression; reduces ERK1/2, Akt/PI3K, EGFR, and Bcl-2 expressions.	[138]
16.	Rosmarinic acid	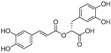	Inhibits microtubule affinity regulating kinase (MARK4); suppresses inflammatory markers COX-2 and TNF-α and the expression of NF-KB; reduces MAPK/ERK and PI3K/Akt signaling pathways; activates caspases and PARP.	[139,140]
17.	Caffeic acidphenethyl ester	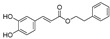	Arrests cell cycle in G1 and G2/M; decreases the phosphorylation of EGFR in different cancer cells; reduces the expression of Akt, Akt1, Akt2, Akt3, phospho-Akt Ser473, phospho-Akt Thr308, GSK3β, FOXO1, FOXO3a, phospho-FOXO1 Thr24, and phospho-FoxO3a Thr32 in Tw2.6 cells.	[118]

**Table 2:** Anticancer effects of some halophytic plants, their mechanism of action, major phytoconstituents, and the IC_50_ values (column 5, µg/mL).

Plant	Location	Active Extract	Main Constituents	Cell Lines/In Vivo Testing, IC_50_ Values	Proposed Mechanism	References
*Anabasis articulata*	Saudi Arabia	Aq. ethanolic extract	Kaempferol 3-neohesperidosid, 6-gingerol, triterpenes, steroidal saponins, and alkaloids	Panc1 (human pancreatic cancer cell line, derived from ductal cell pancreatic carcinoma), IC_50_ 998.5		[70]
	Egypt	Methylene chloride		HePG-2 (human, hepatic carcinoma cell), IC_50_ 6.9; HCT-116, IC_50_ 5.5		[162]
*Arthrocnemum indicum*	Tunisia	Shoot aqueous methanol extract	Gallic acid, 3-hydroxy-4′-methoxyflavone, cyanidin, chrysoeriol, quercetin, catechol, syringic acid, luteolin	Shoot extracts inhibited Caco-2 (human, colorectal adenocarcinoma cells) colon cancer cell growth in a dose-dependent manner	Cell cycle blocking at the G2/M phase	[163]
*Arthrocnemum macrostachyum*	Egypt	Methanol extract	Phenolic acids and flavonoids	In vivo anticancer effect against Ehrlich solid tumor in mice	Increased tissue necrosis and apoptosis, enhanced DNA fragmentation, upregulated cell cycle regulatory genes (*Cdc2* and con*nexin26*), and decreased TNFa levels in tumor tissues	[164]
*Asplenium ceterach*	Bulgaria	Aqueous methanol	Phenolic acids and flavonoids	A549 (human, adenocarcinoma, hypotriploid alveolar basal epithelial cells), FL, HeLa (IC_50_ 40.48)	Strong proapoptotic potential against HeLa (human, cervical cancer cell line)	[165]
*Avicennia marina*	Saudi Arabia	Hexane fraction	Betaine and hymecromone	HCT-116, IC_50_ 23.7; HepG2, IC_50_ 44.9; MCF-7, IC_50_ 79.55	Inhibition of cell cycle in G0/G1 and S phases in HepG2 and MCF-7	[151]
*Carpobrotus edulis*	Portugal	Leaf methanol extract and different fractions.	*β*-amyrin, uvaol, oleanolic acid, monogalactosyl diacylglycerol, catechin, epicatechin, and procyanidin B5	L5178 (mouse, T-cell lymphoma cells), and L5178 (mouse, T-cell lymphoma cells) transfected with pHa MDR1/A retrovirus	Inhibition of P-glycoprotein in MDR1-transfected mouse lymphoma cells	[166]
*Chenopodium formosanum*	Taiwan	Grain extract	Polyphenols and prebiotic dietary fiber	In vivo colon carcinogenesis induced by 1,2-dimethylhydrazine and dextran sulfate sodium in rats	Increase *Bax* and *caspase-9* expressions; reduced TP53 and *Bcl-2* expression; decreased expressions of proliferating cell nuclear antigen and cyclooxygenase-2; regulation of apoptosis-related proteins	[167,168]
*Mesembryanthemum crystallinum*	Korea	Ethanol extracts and its fractions	Phenolics and flavonoids	Inhibition of HCT116 cell growth in dose-dependent manner	Increased G2/M cell population and increased ROS levels in cells	[169]
*Echinophora spinosa*	Italy	Essential oils	*p*-Cymene, *β*-Phellandrene, *β*-Phellandrene, myristicin	U937, IC_50_ 14.5–43.4	Induced apoptosis in U937 cell line (human monocytic cell based)	[170]
*Glaucium flavum*	Tunis	Ethyl acetate extract	Isoquinoline alkaloids, kaempferol, caffeic acid, catechin hydrate, syringic acid, chlorogenic acid, isoquercitrin, and *trans*-hydroxycinnamic acid	MCF-7, IC_50_ 135		[171]
	Algeria	CH_2_Cl_2_ extract		MDA-MB-435, MDA-MB-231, and Hs578T (IC_50_ 7.9–13.6) as well as in vivo tumor chorioallantoic membrane (CAM) model	Hinders angiogenesis, induction of apoptotic processes, and/or limited neovessel formation inside the tumor	[172]
	Iran	Methanol extract and rich alkaloid fraction		HT-29, IC_50_ 22.32 L; Caco-2, IC_50_ 52.38		[173]
*Glehnia littoralis*	Korea	Hexane fraction Aqueous methanol fraction	Furanocoumarin bitter principle and polyacetylene alcohols	HT-29 (77% inhibition at 50 mg/mL extract)	Induced chromatin condensation and nuclear fragmentation, suggesting the presence of apoptotic cells; reduced *mRNA* expression of Bcl-2, cyclooxygenase (COX-2), and inducible nitric oxide synthase (iNOS)	[174]
*Limonium densiflorum*	Tunisia	CHCl_3_ extract	Gallic acid, epigallocatechin, quercitrin, myricetin, dihydrokaempferol, isorhamnetin	A-549, IC_50_ 29 µg/mL; DLD-1, IC_50_ 85)	Isorhamnetin induced apoptosis through activation of peroxisome proliferator-activated receptor γ pathway in gastric cancer	[74,175]
*Limonium bonduelli*	Algeria	*n*-Butanol extract	Flavonoids (eriodictyol, luteolin, apigenin) and 4-hydroxy-3-methoxy benzoic acid; ethyl acetate extract of *L. bonduelli* and pure flavonoids, eriodictyol and luteolin	Dose-dependent growth inhibition of HT-29 and HeLa cell-lines		[176]
*Lotus creticus* L	Portugal	Acetone extract (aerial part) Ethanol extract (fruits)	Steroids, coumarins, tannins, and flavonoids, e.g., catechin, epicatechin, isorhamnetin, quercetin, isorhamnetin-*O*-hexoside, quercetin-*O*-hexoside, myricetin-*O*-hexoside	Extracts had low toxicity RAW 264.7		[177]
*Lycium shawii*	Saudi Arabia	Aqueous ethanol extract	Flavonoids, 3-gluco-7-rhamnosyl quercetin, luteolin 7-*O*-glucoside, kaempferol-3-*O*-glucoside	MCF7, 194.5 µg/mL; K562, 464.9 µg/mL	Induced apoptosis and cell membrane damage due to necrosis and late apoptosis	[70]
*Malcolmia littorea*	Portugal	Polar extracts of flower and roots	Phenolic acids and flavonoids including salicylic acid and luteolin-7-*O*-glucoside.	HepG2 (viability 38.3%) HEK 293 cells (viability 93.1%)		[178]
*Mentha piperita*	China	Essential oils	Menthyl acetate, cineol, menthol, pulegone, and caryophyllene oxide	SPC-A1, IC_50_ 10.89; K562, IC_50_ 16.16; and SGC-7901, IC_50_ 38.76.		[159]
*Pulicaria undulata*	Saudi Arabia	Aqueous ethanolic extract	Flavonoids of kaempferol-, luteolin-, and quercetin-based glycosides	MCF-7, 519.2 µg/mL; K562, 1212 µg/mL; PANC-1, 1535 µg/mL	Cell cycle arrest at the Q1 and Q2 quadrants, and necrosis in late apoptosis	[154]
*Pulicaria crispa*	Saudi Arabia	Aqueous ethanolic extract	Sterols, triterpenoids, essential oils, phenolics, and flavonoids	MDA-MB-231, IC_50_ 180 µg/mL	Loss of cancer cell integrity, shrinkage of cytoplasm, and cell detachment	[179]
*Reaumuria vermiculata*	Tunisia	Hexane and CH_2_Cl_2_	Myricetin, phenolics, and flavonoids	A-549, IC_50_ 17, (hexane extract), and 23 (dichloromethane extract)		[158]
	Egypt	Aqueous methanol extract		Huh-7, IC_50_ 2.4; HCT-116, IC_50_ 1.8; MCF-7, IC_50_ 1.3; PC-3, IC_50_ 1.5		[180]
*Salicornia herbacea*	Korea	Crude and fine polysaccharide	Polysaccharides and phenolic compounds	HT-29	Inhibition of cyclin B1 and Cdc2 *mRNA*G2/M arrest	[181]
*Salvadora persica*	Saudi Arabia	Ethanol extracts of fruits	Essential oils, alkaloids, steroids, cetyl dasycarpidan-1-methanol, tetracosamethyl-cyclododecasiloxane, eicosamethyl-cyclodecasiloxane, and 1-monolinoleoylglycerol	MCF7, IC_50_ 17.50; A2780, IC_50_ 8.35; HT29, IC_50_ 5.12		[182]
*Salvadora persica* L	Egypt	Bark petroleum ether		HepG, IC_50_ 43.6l; MCF-7, IC_50_ 44.3; A549, IC_50_ 19.87 L		[183]
*Suaeda fruticosa*	Pakistan	Methanol and CHCl_3_ extracts	Phenolics, flavonoids, saponins, fatty acids	MCF-7 (63.44% and 45.01% cell viability in methanol and CH_2_Cl_2_ at 200 μg/mL), MDA-MB-231 (77.75% and 67.22% cell viability in methanol and dichloromethane at 200 μg/mL), and DU-145 (62.83% and 25.88% cells viability in methanol and dichloromethane at 200 μg/mL)		[184]
	Tunisia	CH_2_Cl_2_ extract		A-549, IC_50_ 49 ± 7; DLD-1, IC_50_ 10 ± 1; Caco-2, IC_50_ 140 ± 13 µg/mL; HT-29, (IC_50_ 12 ± 14		[157]
	Saudi Arabia	Hexane extract		HCT-116, IC_50_ 17.15; MCF-7, IC_50_ 28.1; HepG2, IC_50_ 33.2	Arrest the cell cycle at the G0-G1 phase	[150]
*Tamarix gallica*	Tunisia	Methanolic extracts	Phenolic acids and flavonoids	Caco-2, 38% inhibition in cell growth at 100 µg/mL	Decreased DNA synthesis, arrested cell mitosis at G2/M phase; changes in the cell-cycle-associated proteins (cyclin B1, p38, Erk1/2, Chk1, and Chk2) correlated with changes in the cell cycle distribution	[185]
	India			Protects against liver carcinogenesis initiated by diethylnitrosamine and 2-acetylaminofluorene	Restoration of cellular antioxidant enzymes, detoxifying enzymes, ODC activity, and DNA synthesis.	[161]
*Zygophyllum album*	Tunisia	CH_2_Cl_2_ extract	Isorhamnetin-3-*O*-rutinoside, quinovic acid derivatives, malvidin 3-rhamnoside, quercetin 3-sulfate	A-549, IC_50_ 37; DLD-1, IC_50_ 48	Downregulation of *cyclin B1* and *cyclin dependent kinase*; upregulation of TP53 and caspase 3	[155]
	Egypt			HepG2 IC_50_ 27.74		[186]
*Zygophyllum coccineum*	Saudi Arabia	Aqueous ethanolic extract	Phenolics, flavonoids, alkaloids, quinovic acid derivatives.	MCF-7, IC_50_ 3.47; HCT-116, IC_50_ 3.19; HepG2, IC_50_ 2.27	Inhibition of human topoisomerase-IIβ	[153]
